# Supplementary material for: Omicron-BA.1 Dispersion Rates in Mexico Varied According to the Regional Epidemic Patterns and the Diversity of Local Delta Subvariants
Source: Viruses. 2023 Jan 15;15(1):243. doi: 10.3390/v15010243 (PMC9863047; doi:10.3390/v15010243)
Supplement: Supplementary file 1 [file viruses-15-00243-s001.zip › viruses-215149_Dec19_SupplementaryFigures.pdf]

Tittle: Omicron-BA.1 dispersion rates in Mexico varied according to the regional epidemic patterns and the diversity of local Delta subvariants

Journal name: *Viruses*

Authors: Selene Zárate, Blanca Taboada, Mauricio Rosales-Rivera, Rodrigo García-López, José Esteban Muñoz-Medina, Alejandro Sanchez-Flores, Alfredo Herrera-Estrella, Bruno Gómez-Gil, Nelly Selem Mojica7, Angel Gustavo Salas-Lais, Joel Armando Vazquez-Perez, David Alejandro Cabrera-Gaytán, Larissa Fernandes-Matano, Luis Antonio Uribe-Noguez, Juan Bautista Chale-Dzul, Brenda Irasema Maldonado Meza, Fidencio Mejía-Nepomuceno, Rogelio Pérez-Padilla, Rosa María Gutiérrez-Ríos, Antonio Loza, Benjamin Roche, Susana López, Carlos F. Arias.

Correspondence: Selene Zárate (selene.zarate@uacm.edu.mx) from Posgrado en Ciencias Genómicas, Universidad Autónoma de la Ciudad de México, Ciudad de México, Mexico.and Blanca Taboada (blanca.taboada@ibt.unam.mx) and Carlos F. Arias (carlos.arias@ibt.unam.mx) from Departamento de Genética del Desarrollo y Fisiología Molecular, Instituto de Biotecnología, Universidad Nacional Autónoma de México, Cuernavaca, Morelos, Mexico.

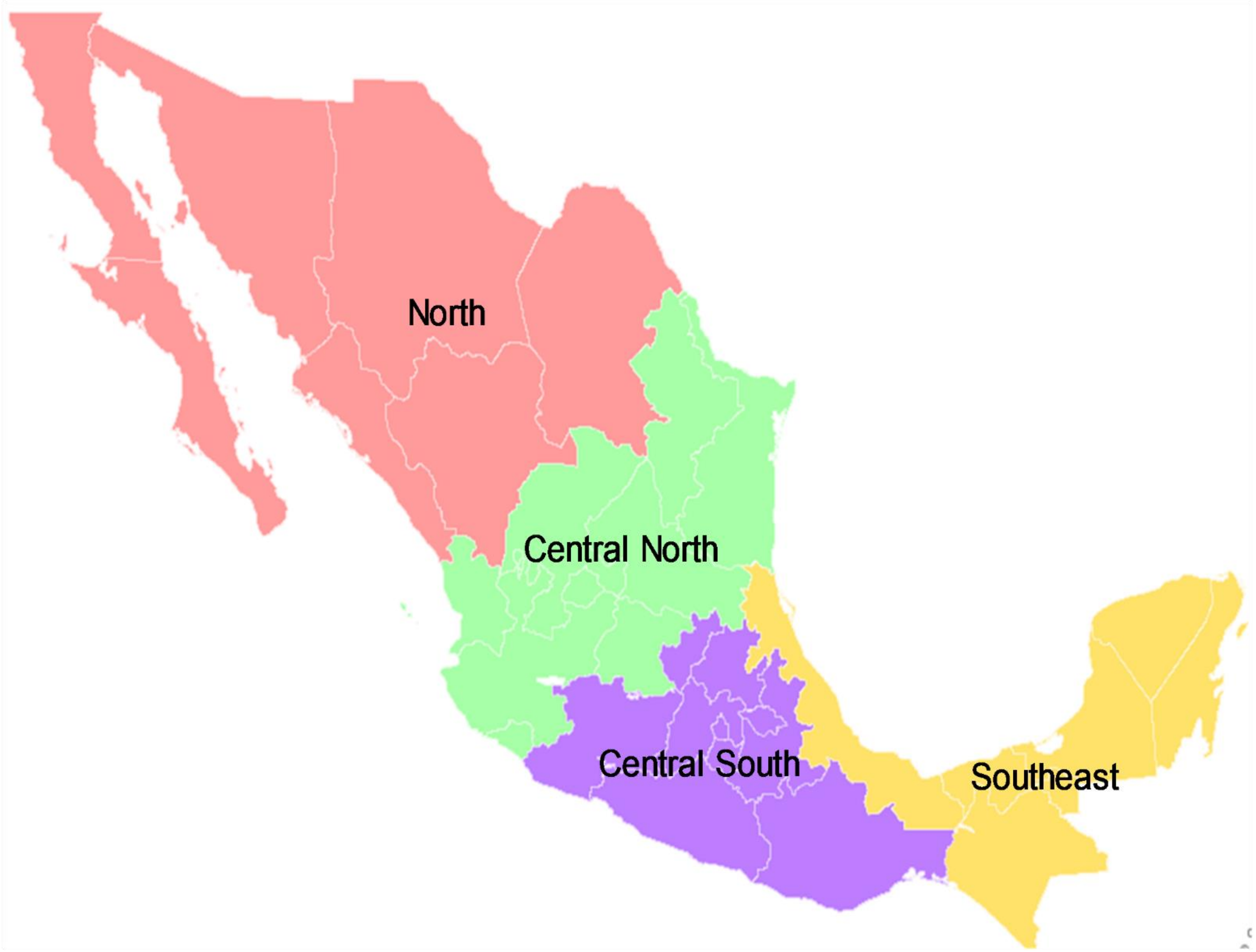

Figure S1. Map of the 32 States in Mexico with the four geographical regions compared in the study.

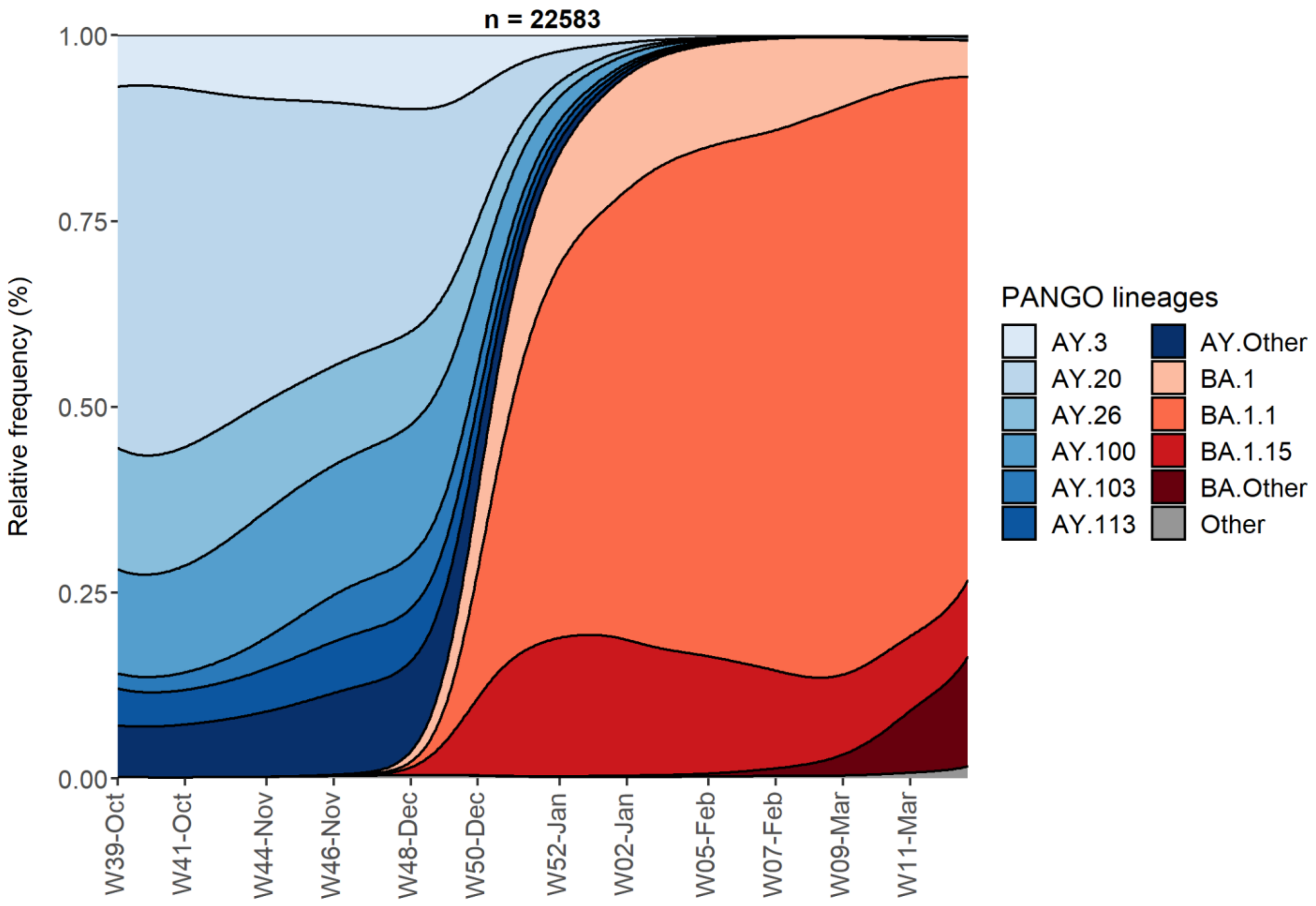

Figure S2. Stacked density plot of the weekly relative frequency of SARS-CoV-2 lineage circulation in Mexico from October 2021 until March 2022

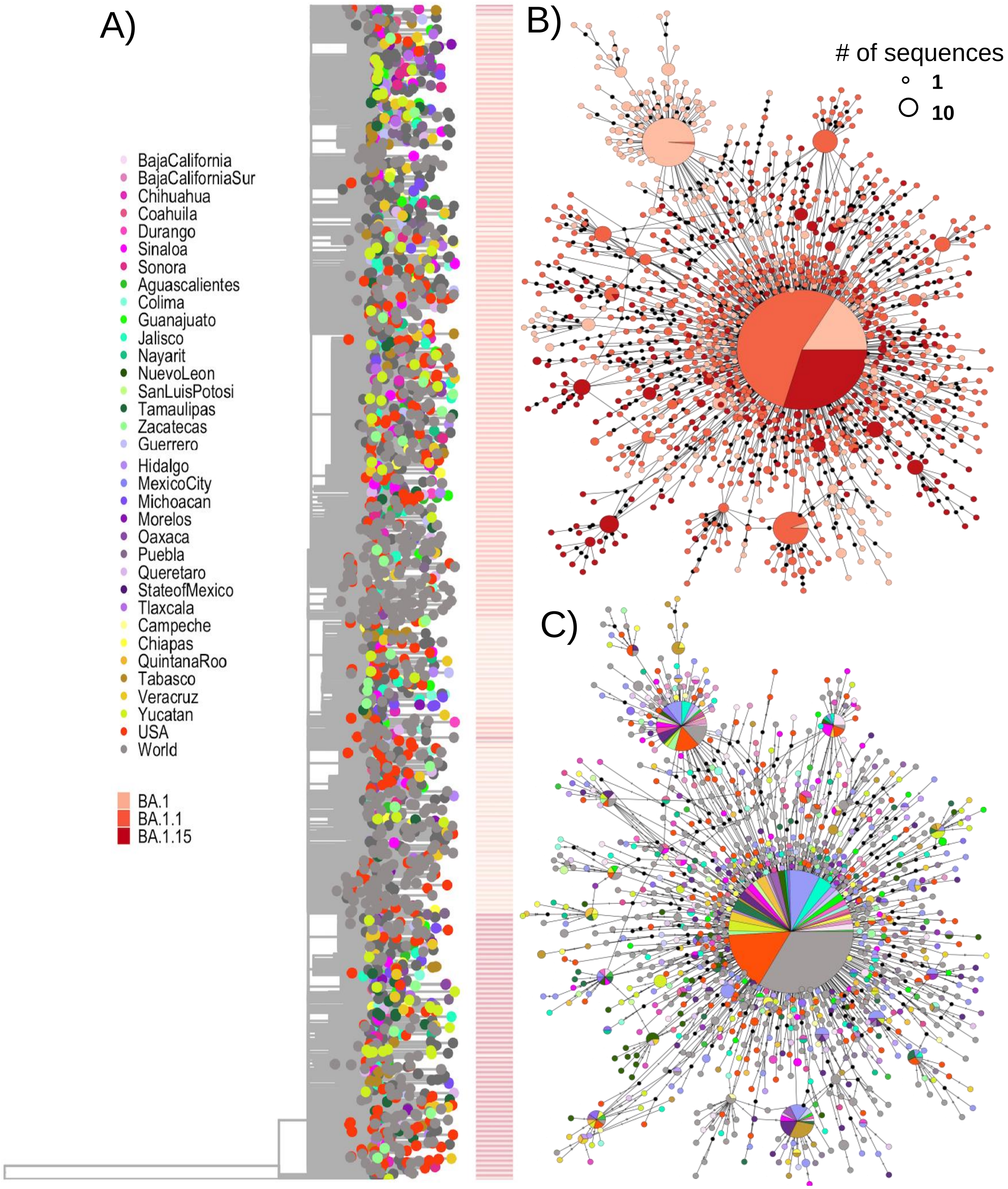

Figure S3. Phylogeny and haplotype network of BA.1, BA.1.1, and BA.1.15 Omicron sublineages. Sequences from these sublineages from Mexico and abroad were used. A) Time-scaled maximum-likelihood phylogeny. The tips are colored by the state of sampling and a heatmap with the sublineage is shown on the side. B and C) Haplotype network colored by sublineage and state of sampling, respectively. The size of the circles corresponds to the number of samples within the same haplotype (scale is provided). The black circles in the haplotype networks represent mutations between sequences.

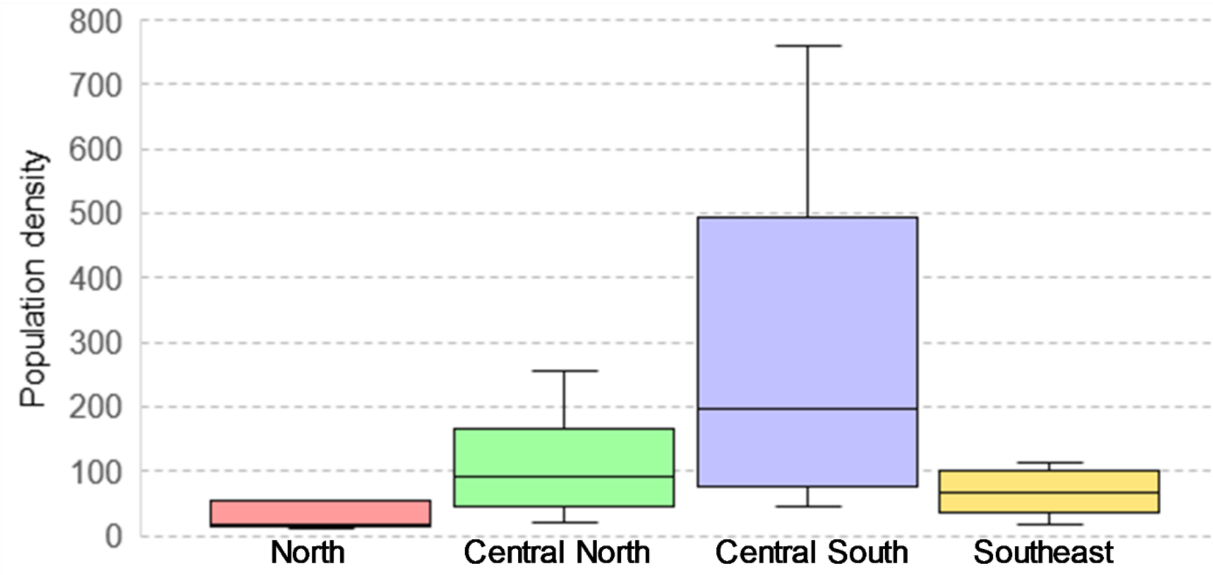

Figure S4. Distributions of the population density in the four regions in Mexico. The density was calculated as inhabitants per square kilometer (0.62 miles). Each box shows the interquartile range (IQR) and the median, whilst the whiskers depend on the kurtosis. Mexico City, with density over 6000, was considered an outlier (further than 1.5 times the IQR from the boxes) and is not shown.
